# Case 4/2019 - Pulmonary Atresia, Ventricular Septal Defect, and Systemic-Pulmonary Collateral Circulation developing after prior Blalock-Taussig, in a Symptomatic 47-Year-Old Adult

**DOI:** 10.5935/abc.20190121

**Published:** 2019-07

**Authors:** Edmar Atik, Maria Angélica Binotto, Alessandra Costa Barreto, Walther Ishikawa

**Affiliations:** Instituto do Coração do Hospital das Clínicas da Faculdade de Medicina da Universidade de São Paulo, São Paulo, SP - Brazil

**Keywords:** Pulmonary Atresia, Heart Septal Defects, Ventricular, Collateral Circulation, Adult, Clinical Evolution

## Clinical data

The patient had no symptoms from birth to young adulthood, when he developed more severe hypoxia, which required a Blalock-Taussig anastomosis on the right. Five years ago, worsening of hypoxia was again observed, with hemapheresis every 6 months, in addition to fatigue on small to medium exertion and with associated ventricular dysfunction. He used f warfarin, carvedilol and enalapril, in addition to nasal oxygen at night.

**Physical examination:** good overall status, eupneic, moderate cyanosis in the extremities, marked digital clubbing, normal pulses in the 4 limbs. Weight: 68 kg, Height: 170 cm, right upper limb blood pressure: 125 x 90 mmHg, HR: 80 bpm, O_2_ saturation = 82%, Hg = 24.5 g / L, Hct = 75%.

**Precordium:** apex beat palpable outside the left hemiclavicular line, with clear systolic impulses at the left sternal border (LSB). Hyperphonetic heart sounds, inconstant split 2^nd^ heart sound, discrete systolic murmur along the LSB, and moderate continuous murmur at the right sternal border and right dorsum. The liver was not palpable, and the lungs were clear.

### Complementary examinations

**Figure 1:**
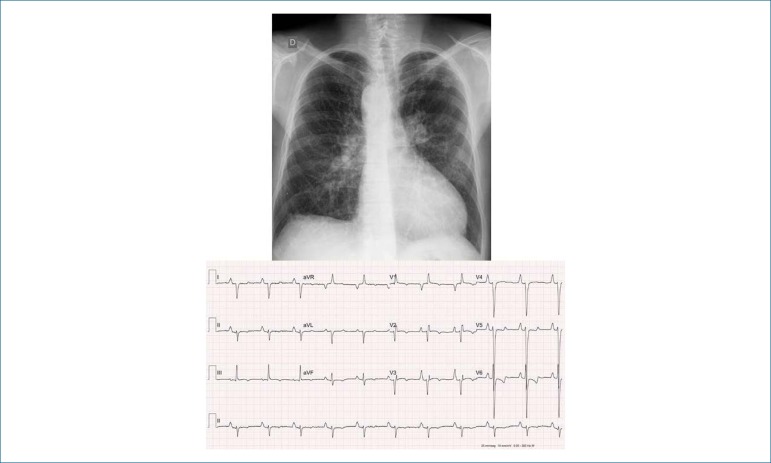
Chest X-ray showing enlarged cardiac area with ventricular dominance and increased pulmonary vascular network. Electrocardiogram shows right cavity overload.

**Electrocardiogram:** Sinus rhythm, signs of right cavity overload, with apiculate P wave and high in the left precordial, and in I and II leads (AP = +40º), and QRS complex showing predominance of S waves from V3 to V6 and axis deviated to the right (AQRS = + 180º). The T wave was negative in the precordial leads (AT = +50º) (Figure 1).

**Chest X-ray:** Discrete enlargement of the cardiac area due to elongated and rounded left ventricular arch and excavated middle arch (CTI = 0.53). The aortic arch is on the right, with an increased pulmonary vascular network in the hila and lower lobes (Figure 1).

**Echocardiogram:** Normal atrioventricular connection, pulmonary atresia and perimembranous 30-mm ventricular septal defect and single outflow tract with aorta (42 mm), overriding the interventricular septum for more than 50%. The right atrium is very dilated (33 cm^2^ area) and the left atrium (38 mm) is discretely dilated. The right ventricle (40 mm) is dilated and hypertrophic, with moderate dysfunction. The left ventricle (40 mm) is hypertrophic with 57% function by the Simpson method. Blalock-Taussig anastomosis was not visualized.

**Myocardial nuclear magnetic resonance imaging:** The diagnosis was confirmed with similar measurements. RVEDV = 120 mL/m^2^ and RV function = 37%. LVEDV = 94 mL/m^2^ with LV function = 54%. Late enhancement was observed in the lower and septal region.

**Figure 2:**
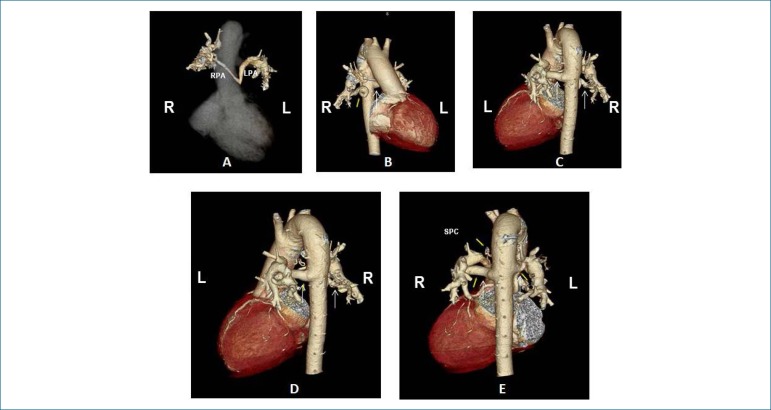
Angiotomography depicting pulmonary artery hypoplasia, especially the right one in A, and the well-developed intraparenchymal pulmonary artery tree due to large systemic-pulmonary collaterals (arrows) on the left and on the right, from the descending aorta in B, C, D, and E , with aortic arch on the right. RPA: right pulmonary artery; LPA: left pulmonary artery; SPC: systemic-pulmonary collateral; R: right; L: left.

**Angiotomography:** It showed marked hypoplasia of the pulmonary arteries, especially to the right and the well-developed intraparenchymal pulmonary artery tree, mainly visible in the lower lobes, due to large systemic-pulmonary collaterals, on the right and left, from the descending aorta, with the aortic arch to the right (Figure 2).

**Holter:** Frequent ventricular extrasystoles (<1% of the total), without supraventricular or ventricular tachycardia.

**Clinical diagnosis:** Pulmonary atresia, ventricular septal defect and systemic-pulmonary collateral circulation with previous Blalock-Taussig occlusion, with right ventricular dysfunction and signs of marked progressive hypoxia in later adulthood.

**Clinical reasoning:** There were clinical elements leading to a diagnosis of congenital heart disease, with arterial malposition considering the hyperphonetic heart sounds and pulmonary atresia in the presence of a continuous clear murmur at the right upper sternal border and right dorsal region, indicating systemic-pulmonary collaterals due to pulmonary perfusion. The right ventricular overload on the electrocardiogram demonstrates the predominance of this ventricle given the correlated hypertrophy. The discrete degree of hypoxia with oxygen saturation of approximately 80% is associated with the increased pulmonary vascular network on the chest X-ray. But even in adults, it provides a considerable increase in red blood cells and their levels in relation to that of serum. The diagnosis of the anomaly was well established by the echocardiography, MRI and mainly by the angiography.

**Differential diagnosis:** Other heart diseases that accompany ventricular septal defect and pulmonary atresia show other features that differentiate them in the usual complementary exams, such as the double inlet right or left ventricle, atrioventricular valve atresia, corrected transposition of the great arteries, and in rarer diseases.

**Conduct:** Despite the balance of the pulmonary and systemic flows over time, with signs of hypoxemia and myocardial dysfunction, the need to increase pulmonary flow to improve quality of life with better physical tolerance is presumed. The consideration of the expectant conduct would not be totally ruled out considering the patient's age, with other development risks.

**Comments:** The natural evolution of this patient until the adult age highlights unfavorable elements, considering that the previous palliative surgery, the Blalock-Taussig anastomosis, occluded spontaneously. Hence, it certainly did not influence this temporal evolution. They are represented in this case by the enlargement of the heart cavities, the right ventricular hypertrophy with RV dysfunction and the pronounced increase in the concentration of red blood cells, which repeatedly required hemapheresis. The doubt persists regarding the performance of another systemic-pulmonary anastomosis to further prolong life and improve quality of life. On the other hand, the expectant conduct should also be considered in this case, given the high and considerable surgical risk in this age group.^1^

The question is, in similar cases in childhood, whether it would be more convenient to attempt an earlier correction. Undoubtedly, it should always be considered at different times to create an anatomic shape that is adequate and favorable to blood dynamics.^2^
